# Medical News and Miscellany

**Published:** 1898-01

**Authors:** 


### Medical News and Miscellany.

---

**Dr. A. Howell** has located at Sunny Lane, Texas.

---

**A Happy New Year** to all the readers of the Red-Back.

---

**Married.**—At Hearne, Tex., Dec. 21, ult., Dr. Hatch Winfield Cummings to Miss Pauline Eckerle, all of that city.

---

**Dr. W. H. Lancaster,** formerly of Moulton, Texas, has located at Waco. The doctor has the best wishes of the Journal.

---

**Dr. Edward B. Jackson**, of Houston, Texas, desires a young physician partner. Must be sober and energetic. Write or call for particulars.

---

**Dr. J. J. Burroughs,** a long time resident of Houston, and a surgeon with a national reputation, has removed to Monterey, Mexico. Dr. Burroughs will be a great loss to Houston and to the Texas medical profession.

---

**Dr. B. F. Church,** the well known oculist of Dallas, has removed to Los Angeles, California. We commend the doctor to the guild and to the people as worthy of their confidence and esteem. He is a skilled oculist, and a regular, in full fellowship with the Texas profession.

---

**Dr. Harvey Reed,** having accepted the position of Superintendent and Surgeon in Charge of Wyoming General Hospital, Rock Springs, Wyoming, has resigned his position as editor of the *Columbus Medical Journal.* Dr. J. E. Brown succeeds him as editor and manager.

---

**Polk's Medical and Surgical Register of the United States.**—We are pleased to announce that a new edition of

"Polks Register" is now in course of preparation. This Register has become a necessity with the medical profession, and we feel assured the news of the new edition will be gladly received.

---

**Wanted.**—To purchase a practice in some railroad town in Texas. A good location off railroad not objected to. Please write, describing your location, value of practice annually, and number of inhabitants in town. Would buy residence property to get good location. South Texas preferred. Address,

J. R. GEORGE, M. D.,
Corrigan, Texas.

---

**The Central Texas Medical Association** will hold its next semi-annual meeting at Hillsboro, Texas, Tuesday, January 11 (inst.). A splendid program has been arranged for the occasion. Dr. W. C. Blailock, President, will hold a surgical clinic at the close of the surgical section, and physicians having difficult cases "will find this an excellent opportunity to present them" for examination and discussion. Program received too late for publication in full.

---

**At the** last meeting of the Austin District Medical Society, which was held in Austin on December 16, 1897, the following officers were elected for the ensuing year: President, Dr. W. T. Richmond, of Manor; First Vice-President, Dr. T. O. Maxwell, of Austin; Second Vice-President, Dr. A. Garwood, of New Braunfels; Secretary and Treasurer, Dr. S. E. Hudson, of Austin; on the Board of Censors, Dr. Joe S. Wooten, of Austin, Dr. J. J. Jones, of Austin, and Dr. J. Angus Gillis, of Buda.

